# Elevation of transaminases after MMP® session with methotrexate for alopecia areata treatment – how much do we know about the risks of systemic absorption of the technique?^☆^

**DOI:** 10.1016/j.abd.2021.01.013

**Published:** 2022-12-27

**Authors:** Bianca Lopes Nogueira, Renan Rangel Bonamigo, Renata Heck

**Affiliations:** a Dermatology Service, Ambulatory of Sanitary Dermatology, Secretaria de Saúde do Estado do Rio Grande do Sul (SES/RS), Porto Alegre, RS, Brazil; b Faculty of Medicine, Universidade Federal do Rio Grande do Sul (UFRGS), Porto Alegre, RS, Brazil; c Dermatology Service, Universidade Federal de Ciências da Saúde de Porto Alegre, Porto Alegre, RS, Brazil

Dear Editor,

There is a higher incidence of alopecia areata (AA) in patients with lupus erythematosus.^1^ Some cases of AA are refractory to the established treatments. Percutaneous drug induction techniques have shown promising results,^2^ including the microinfusion of drugs into the skin (MMP®, *Microinfusão de Medicamentos na Pele*).^3^ Given its recent use, little is known about its safety. This case report describes a case of transaminase elevation after a session of MMP® with methotrexate (MTX) in a patient with AA associated with systemic lupus erythematosus (SLE).

A 37-year-old white female patient, diagnosed with SLE 13 years ago, with good clinical and laboratory control, using hydroxychloroquine and dapsone was assessed. She had an ophiasis pattern AA, with yellow and black dots and exclamation mark hairs on dermoscopy. Histopathological examination showed peribulbar lymphocytic infiltrate and miniaturized follicles, excluding the diagnosis of associated lupus alopecia. She underwent topical treatments with no response but showed temporary improvement of the alopecia while receiving MTX15 mg/week for the treatment of SLE, discontinued after nine months due to hepatotoxicity. Two years later, it was decided to employ the transepidermal use of this medication, using the MMP® technique.

Exams prior to the procedure showed no changes in blood count, GOT of 26 U/L, GPT of 20 U/L, Gamma-GT of 14 U/L, alkaline phosphatase of 57 U/L, and total bilirubin of 0.45 mg/dL (Direct 0.18 mg /dL ‒ Indirect 0.27 mg/dL). She underwent an MMP® session with the microinfusion of 20 mg of MTX in the alopecia patches. Control exams after one week showed GOT of 37 U/L and GPT of 48 U/L. She was an asymptomatic patient, and did not use any other medications, without alcohol consumption or possible confounding factors. A new control exam was performed after two weeks which revealed that the transaminases had returned to baseline parameters. Treatment was discontinued.

The MMP® technique consists of the percutaneous administration of drugs using a tattoo device. Its needles allow drug infusion regardless of the molecular weight, chemical characteristics of the medium (lipophilic or hydrophilic), bleeding, or exudation.^3^ Its use has been described in the treatment of idiopathic guttate hypomelanosis, androgenetic alopecia, and psoriasis.^3^

Other transcutaneous permeation techniques, such as fractional laser, microneedling, radiofrequency, sonophoresis, and iontophoresis have been discussed with promising results.^2^

The use of systemic MTX for cases of refractory AA has shown satisfactory results.^4^ However, it carries a risk of myelosuppression and hepatotoxicity.^4^, ^5^ The first description of its use with the MMP® technique referred to patients with psoriasis who were intolerant to systemic therapy, with good response and tolerability.^5^ The advantages of this method include reduced toxicity by avoiding the hepatic first-pass metabolism, and the use of lower doses of medication due to good permeation into the dermis.^5^

The authors chose this technique due to the lower systemic absorption, aiming to prevent the previously reported hepatic adverse effects. However, after just one session of MMP® on the scalp, a highly vascularized region, an elevation of transaminases was identified, which proved to be transient. It is important to note that if the treatment had been continued, a probable worsening of transaminase elevation would have been observed.

The authors emphasize the importance of the rational use of techniques that involve the permeation of drugs through the skin, taking into consideration the possibility of absorption and systemic adverse effects. As it is an off-label medication for AA, with the transdermal route not being described in the package insert, the authors suggest a cautious use of the drug, with laboratory monitoring and regular clinical evaluation.

## Financial support

None declared.

## Authors' contributions

Bianca Lopes Nogueira: Design and planning of the study; collection, analysis and interpretation of data; drafting and editing of the manuscript and critical review of important intellectual content; critical review of the literature; approval of the final version of the manuscript.

Renan Rangel Bonamigo: Drafting and editing of the manuscript and critical review of important intellectual content; effective participation in research orientation; intellectual participation in the propaedeutic and/or therapeutic conduct of the studied cases; critical review of the literature; approval of the final version of the manuscript.

Renata Heck: Design and planning of the study; drafting and editing of the manuscript and critical review of important intellectual content; effective participation in research orientation; intellectual participation in the propaedeutic and/or therapeutic conduct of the studied cases; critical review of the literature; approval of the final version of the manuscript.

## Conflicts of interest

None declared.
